# Integrating mental health into primary care for displaced populations: the experience of Mindanao, Philippines

**DOI:** 10.1186/1752-1505-5-3

**Published:** 2011-03-07

**Authors:** Yolanda Mueller, Susanna Cristofani, Carmen Rodriguez, Rohani T Malaguiok, Tatiana Gil, Rebecca F Grais, Renato Souza

**Affiliations:** 1 Epicentre, 8 rue Saint Sabin, 75011 Paris, France; 2 Médecins Sans Frontières, rue de Lausanne 78, CP 116, 1211 Geneva 21, Switzerland; 3 Médecins Sans Frontières, N°01 Manara st, Rosary Heights 10, Cotabato city 9600 Mindanao, Philippines

## Abstract

**Background:**

For more than forty years, episodes of violence in the Mindanao conflict have recurrently led to civilian displacement. In 2008, Medecins Sans Frontieres set up a mental health program integrated into primary health care in Mindanao Region. In this article, we describe a model of mental health care and the characteristics and outcomes of patients attending mental health services.

**Methods:**

Psychologists working in mobile clinics assessed patients referred by trained clinicians located at primary level. They provided psychological first aid, brief psychotherapy and referral for severe patients. Patient characteristics and outcomes in terms of Self-Reporting Questionnaire (SRQ20) and Global Assessment of Functioning score (GAF) are described.

**Results:**

Among the 463 adult patients diagnosed with a common mental disorder with at least two visits, median SRQ20 score diminished from 7 to 3 (p < 0.001) and median GAF score increased from 60 to 70 (p < 0.001). Baseline score and score at last assessment were different for both discharged patients and defaulters (p < 0.001).

**Conclusions:**

Brief psychotherapy sessions provided at primary level during emergencies can potentially improve patients' symptoms of distress.

## Background

During the acute phase of an emergency, mental health interventions to reduce traumatic stress are often put in place. In addition to syndromes often associated with conflict such as post-traumatic stress disorders [1], other disorders also occur, such as depressive or anxiety disorders [2]. Further, in a context of limited access to health care, patients with mental health or neurological disorders not directly linked to the conflict, such as psychosis or epilepsy, may be neglected by vertical interventions related to the conflict or natural disaster [3]. Descriptions of treatment models and research about the outcome of interventions in emergencies are rare [4]. Much of the existing research focuses on post-traumatic disorders, often to the exclusion of other disorders. Less attention may be given to the needs of those with disorders unrelated to the conflict. Vertical trauma-focused services are often juxtaposed against the importance of the integration of trauma-focused care and the treatment of pre-existing mental disorders into general mental health and primary care [5].

Humanitarian organizations now recommend that psychological first aid be provided as part of medical care for victims of violence or natural disasters and that care for people with severe mental illness is integrated into primary health care due to the extreme vulnerability of such patients [4,6,7]. Medecins Sans Frontieres (MSF) has integrated mental health into medical activities in order to respond to mental health needs of people with common and severe mental disorders [3]. Following international recommendations [7], MSF developed a model for mental health care provision where psychological first aid and brief psychotherapy is provided to patients with common mental disorders by trained psychologists working at primary health care level. The diagnosis and treatment of severe mental illness are either provided through a referral system to existing psychiatric care structures or directly if no such structures exist. Here, we describe a model of mental health care adapted to protracted conflicts and the characteristics and outcomes of patients attending mental health services. We discuss lessons learned and the need for continued research on mental health in humanitarian emergencies.

## Methods

### Setting

The Mindanao conflict in the Philippines first flared in the 1960s when the Moros, the Muslim minority, began an armed struggle to regain their ancestral homeland in the southern island [8]. Since then, periods of peace have alternated with periods of short but ferocious clashes between the Bangsamoro rebel forces and the Armed Forces of the Philippines (AFP), displacing tens of thousands of civilians. In August 2008, the peace agreement between the Government of the Philippines (GRP) and the Moro Islamic Liberation Front (MILF) disintegrated and an estimated 700,000 persons were displaced [8]. Most of the fighting between the government and MILF secessionist group took place in the Autonomous Region of Muslim Mindanao (ARMM).

During that time, many had to evacuate under fire, saw their homes destroyed, or witnessed people being wounded or killed. Since, some displaced returned to their homes, facing the risks associated with shelling and fighting during the night. By December 2009, 125 278 people were still estimated to be internally displaced in Central Mindanao [9]. These informal settlement sites, called evacuation centers, were made of local material and plastic sheeting and located in public spaces and on roadsides. Some centers were transformed into semi-permanent resettlement areas because of the persistence of the armed conflict in the home communities of the displaced population. In these confined spaces, the population still encountered fighting and the surrounding presence of armed forces. Relatives in the community hosted nearly half of the displaced.

MSF started to work in Mindanao in November 2008, with the aim of ensuring medical care for the displaced population. Within this framework, the organization set up activities with the authorization of the Ministry of Health. At primary health care level, mobile clinics provided curative and preventive care at the level of the evacuation centres. In addition, the Ministry-of-Health-supported Rural Health Units received additional support in terms of medical supplies, human resources and logistics. Secondary level care was supported by establishing a referral system to the regional hospital. All individuals, whether displaced or members of the host community were eligible to receive care provided free of charge.

### Mental health intervention

**Figure 1:**
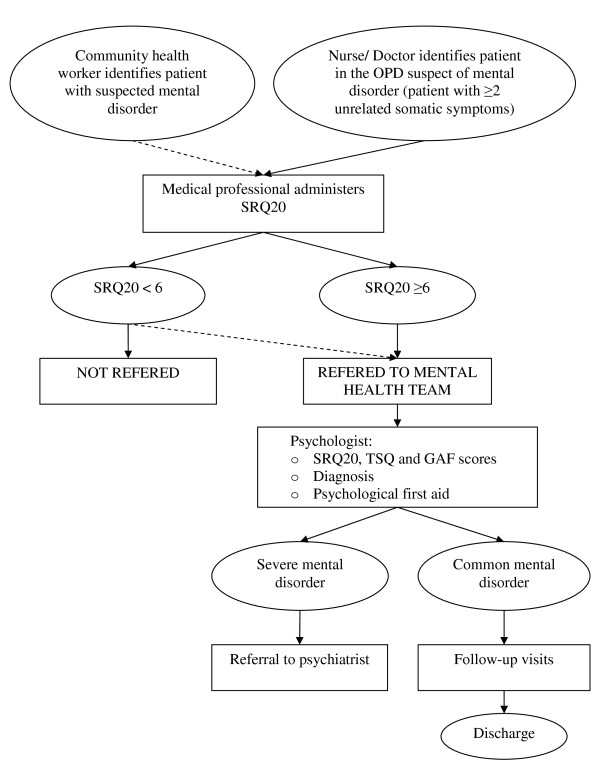
**Model of mental health care delivery in the Médecins Sans Frontières project, Mindanao, Philippines, March-December 2009**. OPD: Outpatient department; SRQ: self-reporting questionnaire; TSQ: Trauma scale questionnaire; GAF: Global Assessment of Functioning

At the community level, community health workers (CHW) were trained by the psychologists to identify and refer cases of mental disorders and epilepsy to the MSF mobile clinics, where the mental health team provided proper diagnosis and treatment (Figure 1). The mental health team consisted of three national psychologists, one national psychologist supervisor, and one expatriate psychologist coordinating the team. At the rural health unit level and in mobile clinics, medical and paramedical staff were trained to suspect potential mental health disorders when faced with a patient presenting with at least two medically unexplained physical symptoms (MUPS). In this case, they performed the self-reporting questionnaire (SRQ20)[10]. In the absence of a cut off score validated for the local population and due to the impossibility to conduct such studies during a humanitarian emergency, we applied a cut off score of equal or superior to six based on the results of a previous study conducted in the same region [11]. Identified patients were then referred to the mental health team. If the score was below six, the patient was usually not referred, except in the presence of other symptoms and signs that led the clinician to consider the patient still in need of mental health support. The mental health team filled the SRQ20 again, to corroborate the score done by the medical staff. The Trauma Scale Questionnaire (TSQ) was used to detect post-traumatic stress disorder [12-14]. Subsequently, the Global Assessment of Functioning score (GAF) was administered in order to assess levels of disability. The psychologist, after making a diagnosis, also provided psychological first aid and structured psychotherapy. All patients were advised to come for follow-up consultations with the mental health team. Patients that did not present to follow-up consultations were reminded to do so by the CHW covering their area. The CHW also collected information about the reason of the default through the community.

Within this model, psychologists located at primary health care level provided psychological first aid and structured psychotherapy to people with common mental disorders [15-18]. Brief psychotherapy sessions consisted of psychoeducation, breathing and relaxation exercises, problem solving counseling and cognitive behavioral techniques for the management of anxiety and depressive symptoms. This choice of psychotherapeutic interventions was based on the existing evidence of its effectiveness and feasibility in primary health care settings in low-income countries [4]. The first follow-up visit was usually planned after 1 week, and from then on every second week. The usual treatment plan consisted of three to four follow-up consultations, although it was possible to add more sessions, taking into consideration the evolution of symptoms of the individual patient. The primary health care psychologists also assessed cases of severe mental illness, before referring them to a psychiatrist working at the secondary level. MSF covered all transportation and psychiatric treatment costs and for referred patient for a minimum of 6 months up to two years of treatment.

### Scores

The self-reporting questionnaire (SRQ20) is a scoring system used to assess levels of distress. It has been endorsed by WHO to be used in primary health care settings for detection of probable cases of mental health disorders. The SRQ20 includes 20 items related to somatic signs, depressive/anxiety factors, and a more cognitive/decreased energy factor [10]. It has been used previously in the Philippines in a population-based survey about the impact on mental health of partner violence [19]. The SRQ20 has also been used in conjunction with other scales to asses outcome of patients undergoing psychotherapy in Brazil [20]. The final score of an individual patient can vary between 0 (no distress) to 20 (maximum distress).

The Global Assessment of Functioning scale (GAF) is a widely used scale that measures overall levels of functionality of an individual. It corresponds to the fifth axis used to organize mental health diagnoses in the Diagnostic and Statistical Manual of Mental Disorders (DSM) [21]. The scale ranges from 01-10 ("persistent danger of severely hurting self or others OR persistent inability to maintain minimum personal hygiene OR serious suicidal act with clear expectation of death") to 91-100 ("superior functioning in a wide range of activities, life's problems never seem to get out of hand, is sought out by others because of his or her many qualities. No symptoms"). For simplification purposes, categories 01-10 are reported in this article as 10, 11-20 as 20, 21-30 as 30, etc.

### Data Analysis

Data were collected by trained psychologists for all patients referred to the mental health team. At each patient's first consultation, information about socio-demographical characteristics, the experienced traumatic events, and syndromic mental health diagnosis was collected. The same scoring system was used at every subsequent visit to evaluate the patients. Translation of the instruments from English to the local language was performed using standard cross-cultural procedures [22]. The supervising psychologist entered the data into an MS Excel spreadsheet (Microsoft, Seattle, Washington). Retrospective analysis of the data was performed using Stata 9 statistical software (Stata Corporation, College Station, Texas). Analysis of outcomes focused on patients over 15 years of age with common mental disorders, in order to have a homogenous group of patients. Scores between first and last visit were compared using the Wilcoxon rank test.

### Ethical considerations

We used routine monitoring data from the MSF program, which was conducted in coordination with the Ministry of Health via a memorandum of understanding, which is the usual procedure for NGOs operating in these contexts. No supplementary interventions were conducted for the analysis presented here. All electronic data were entered anonymously and identifiers were coded. No ethnic or identifying information was entered.

## Results

Between March 4 and December 15 2009, the mental health team assessed 962 patients, totaling 2,242 visits. The mean age of patients was 35 years (SD 15 years). The male:female sex ratio was 1:3.9 for patients over 15 years. Out of the 962 patients referred to the team, 771 (80.1%) were considered to suffer from a mental health disorder after evaluation by the primary health care psychologist (Table 1). The remaining patients consisted either of persons referred to the mental health team for counseling for sexually transmitted infections or patients that were not judged to suffer from a mental disorder after assessment by the psychologist, although initially suspected by the medical teams.

**Table 1:** Type of mental health disorder among 962 patients referred to the mental health team in Mindanao, Philippines, March-December 2009

	Age group			
Type of disorder	0 to 15 years	over 15 years	Missing age	Total
Common mental disorder*	73	661	1	735
Severe mental disorder^+^	3	21	0	24
Child/adolescent mental disorder	11	1	0	12
Others	14	177	0	191
Total	101	860	1	962

* Common mental disorders (CMD): generalized anxiety disorder, depression, post-traumatic stress disorder, acute stress reaction, CMD otherwise specified

^+ ^Severe mental disorders (SMD): schizophrenia, epilepsy, severe depression, psychosis, SMD not otherwise specified

Source: MSF

**Figure 2:**
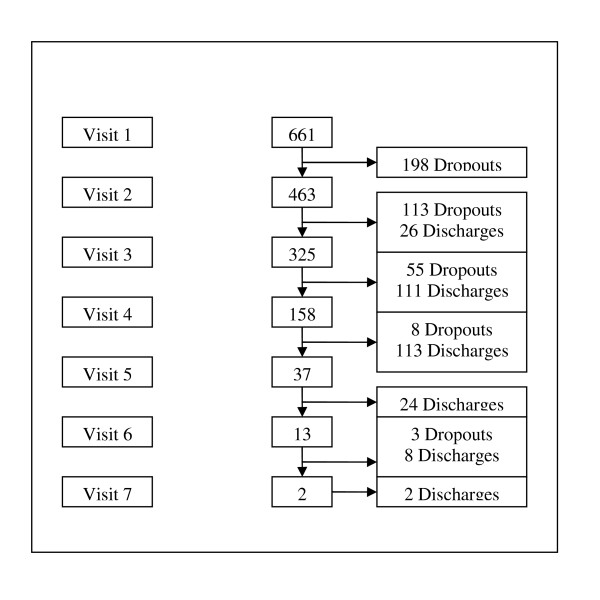
**Flowchart of patients with common mental disorders in the mental health project, Mindanao, Philippines, March-December 2009**. Source: MSF

This paper focuses on the description and outcomes of patients aged over 15 years old and diagnosed with a common mental disorder. The majority of these patients (96%) experienced some traumatic event; the most frequently reported being evacuation of the home in a dangerous situation (54%), experiencing a combat situation (26%) or destruction of property (5%) (Table 2). Furthermore, 11% of the patients reported a death due to violence in the household. Four hundred and sixty-three patients (70%) were seen more than once (Figure 2). Median delay between the first and second visit was 14 days (IQR 7,28), and between subsequent visits ranged between 21 and 28 days. Over half (57%) of the patients did not come back for a scheduled visit (dropouts) before being discharged by the team. Data collected by the CHW showed that 35 to 40% of the dropouts had moved to another location or went back home.

**Table 2:** Characteristics of 661 patients over 15 years old with common mental disorder, Mindanao, Philippines, March-December 2009

	n	%
Age (mean; SD)	39.6 (12.6)	
Sex		
- Female	552	83.5%
- Male	109	16.5%
Marital status:		
- Single	78	11.8%
- Married	472	71.4%
- Divorced	22	3.3%
- Widowed	88	13.3%
Status:		
- Displaced	621	93.9%
- Non-displaced	39	5.9%
Religion:		
- Muslim	657	99.4%
- Christian	4	0.7%
Education:		
- No education	345	52.2%
- Primary	220	33.3%
- Secondary	70	10.6%
- University	24	3.6%
Support:		
- Family/self	597	90.3%
- External aid	62	9.4%
Sleep:		
- With parents	39	5.9%
- With relatives	21	3.2%
- In shelter	598	90.5%
- In the street	2	0.3%
Traumatic event:		
- Evacuation under danger situation	356	53.9%
- Combat situation	171	25.9%
- Destruction of property	31	4.7%
- Witnessing killings	6	0.9%
- Lack of shelter	5	0.8%
- Relative seriously injured	4	0.6%
- Lack of food/water	3	0.5%
- Illness without medical care	3	0.5%
- Witnessing humiliation	2	0.3%
- Beating	1	0.2%
- Torture	1	0.2%
- Physical injury due to combat	1	0.2%
- Others	49	7.4%
Any event	633	95.8%
Any death due to violence in the household	74	11.2%
Any death due to disease in the household	159	24.1%
Any missing household members	18	2.7%

Source: MSF

**Figure 3:**
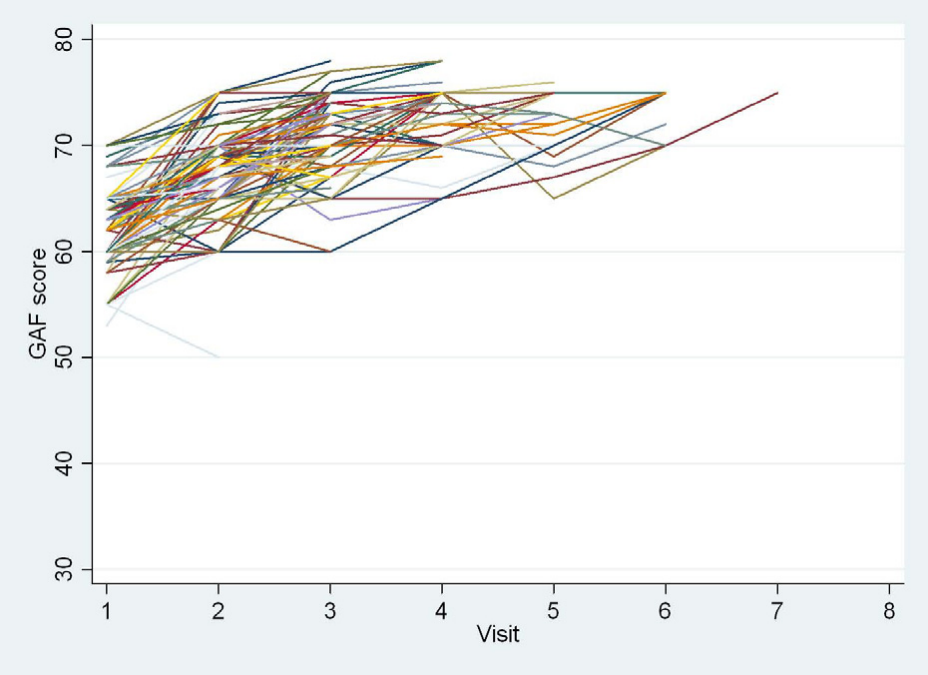
**Evolution of the Global Assessment of Functioning (GAF) scores of 463 patients aged over 15 years with common mental disorders and at least two visits to the mental health project, Mindanao, Philippines, March-December 2009**. One line represents one patient. Source: MSF.

**Figure 4:**
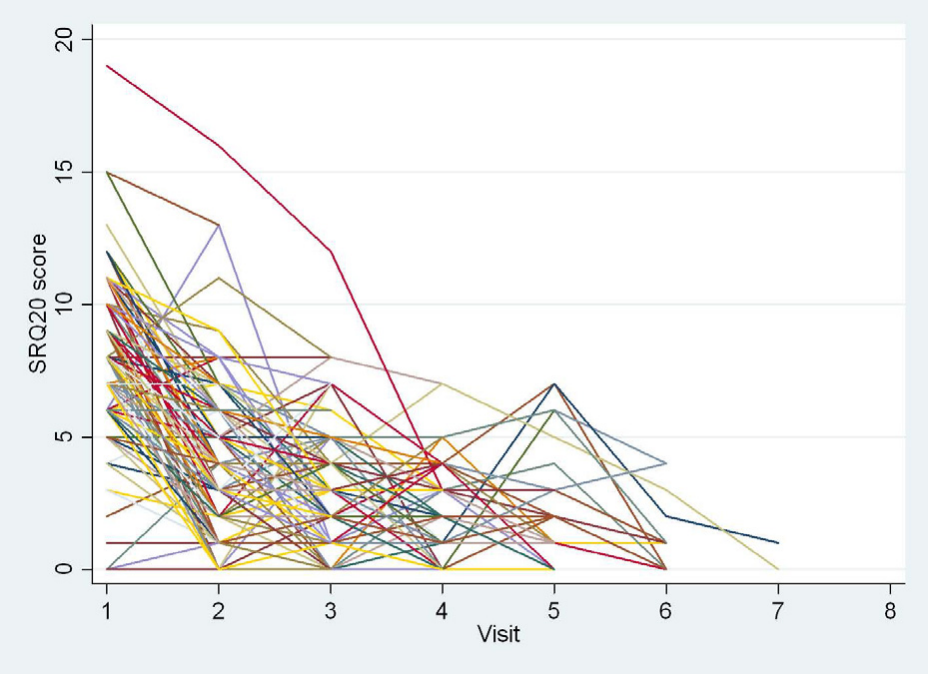
**Evolution of the Self-Reporting Questionnaires (SRQ20) scores of 463 patients aged over 15 years with common mental disorders and at least two visits to the mental health project, Mindanao, Philippines, March-December 2009**. One line represents one patient. Source: MSF

We examined the evolution of the patients at consecutive visits according to the scores described above. Figures 3 and 4 shows the evolution of the individual patients on respectively the GAF and the SRQ20 score, for patients with at least two visits. Between first and last visit, median GAF score increased from 60 (IQR 60, 60) to 70 (IQR 64, 75; Wilcoxon rank test p < 0.001) and median SRQ20 score diminished from 7 (IQR 6,8) to 3 (IQR 1,7; Wilcoxon rank test p < 0.001). The difference between baseline score and score at last assessment was significant for both discharged patients and defaulters (p < 0.001). By analyzing the data (excluding the dropouts) we observed that 46% of the patients had sufficiently improved to allow discharge by the 3rd visit and 87% by the 4^th^.

## Discussion

The Mindanao project in the Philippines shows that simple mental health approaches such as psychological first aid and brief psychotherapy can be integrated into primary health care in an emergency humanitarian context. Furthermore, retrospective analysis of patient data suggest that brief psychotherapy sessions provided at primary level to patients with common mental disorders can potentially improve patients' symptoms of distress, within a few sessions.

Although there were a high number of dropouts from the program, it is important to note that patients did improve before they dropped out. This high proportion of dropouts could be linked to the volatile security context and regular displacements occurring in this population, which may prevent patients from attending consultations. We do not think that this reflects failure of care. Flexibility in the pattern of follow-up is a necessity in such an unstable environment, where regular attendance to appointments at fixed points in time cannot be expected. However, our data show that even a brief and sometimes irregular intervention can lead to substantial improvements in patients' conditions.

Whereas other case series conducted in violent contexts such as Darfur [3], Palestine [23] and Colombia [2] have already described characteristics of patients affected by mental disorders, our data have the advantage of having used standardized outcome measures and not only psychologist's opinion. Interestingly, our series consisted of a higher proportions of patients with common mental disorders when compared to the patients in Darfur [3], which showed a high proportion of severe disorders. This may be a reflection of the active case detection approach used in Mindanao, integrated into primary care, which allowed for detection of non-severe cases of mental disorders.

The creation of a strong network of community health workers was crucial to identify potential patients and to ensure good follow-up. CHWs also played an important role for adherence to psychological support and pharmacological treatment, by speaking with the patient about the importance of finishing treatment. Indeed, without the work done by the CHWs in this project, the proportion of defaulters would probably have been much higher. It was also important to find local psychologists able to speak and understand local languages and cultural issues. This gave patients the opportunity to express themselves in their own language, while receiving professional care from someone coming from the same cultural background. The good collaboration between the medical staff and the mental health team was also an important factor of success of the project. This was facilitated by previous sensitization and training of medical team on mental health issues.

It is worth noting that changes on median GAF scores reflected a progression from moderate symptoms to mild symptoms and good functionality. Although the GAF score has been used previously to measure patient outcome, the SRQ20 score was not validated as such for this purpose. However, we do find this scale useful in this situation, as it is referring to items related to distress not directly related to a specific diagnosis. Besides, it has been used in a number of different cultural contexts. Interestingly, GAF and SRQ scores showed a linear relationship in our dataset (regression coefficient -1.5; 95%CI -1.53, -1.41; p < 0.001), which strengthens our conclusions. Clinicians (doctors and nurses) also judged the SRQ20 to be a useful tool to perform screening of a suspected case before referring them for specialized assessment. Further research on the development and use of outcome measures that can be standardized, acceptable to primary health care practitioners and feasible for routine use in humanitarian settings is of the utmost importance [24].

One of the limitations of this work is the absence of a control group. Indeed, we cannot exclude that the positive outcomes seen in this project are not due to the intervention, but may only reflect the healing effect of time itself. The possibility of bias due to the fact that professionals providing mental health services were the same ones that measured outcome scores can not be excluded. We tried to minimize this by implementing continuous training and quality control on the use of the scales. Further, outside of a study context, inclusion criteria into the program were not strictly defined, allowing for the follow-up of some very paucisymptomatic cases. This inclusion of patients with light symptoms may have accentuated the positive impact of the intervention. This highlights the need for continued formal research in this area.

## Conclusions

This project shows the feasibility and success of implementing mental health care into primary care, as recommended by WHO, even in an unstable context with a mobile population. Brief psychotherapy sessions provided at primary level during emergencies can potentially improve patients' symptoms of distress. The key to success in this project lies in the flexibility given by the mobile set-up, the integration of psychologists as part of the mobile clinic teams, the good network of CHW specifically trained in the identification and follow up of mental health patients, as well as the good collaboration between medical and mental health teams. This multidisciplinary approach should be promoted and widely applied in other humanitarian contexts.

## Competing interests

The authors declare that they have no competing interests.

## Authors' contributions

YM analyzed the data and drafted the manuscript. CR, TG, RTM and SC conceived the data collection system, and contributed to the data interpretation and the revision of the manuscript. RFG and RS made substantial contributions to the data analysis and to the revision of the manuscript. All authors read and approved the final manuscript.
